# Significance of STAT3 in Immune Infiltration and Drug Response in Cancer

**DOI:** 10.3390/biom10060834

**Published:** 2020-05-29

**Authors:** Wei Chen, Xiaoshuo Dai, Yihuan Chen, Fang Tian, Yanyan Zhang, Qiushuang Zhang, Jing Lu

**Affiliations:** 1 Department of Pathophysiology, School of Basic Medical Sciences, Zhengzhou University, Zhengzhou 450001, Henan, China; chenweihaley@gs.zzu.edu.cn (W.C.); daixiaoshuo@gs.zzu.edu.cn (X.D.); chenyihuan98@gs.zzu.edu.cn (Y.C.); tianfang@zzu.edu.cn (F.T.); zhangyanyan@zzu.edu.cn (Y.Z.); zhangqiushuang@gs.zzu.edu.cn (Q.Z.); 2 Collaborative Innovation Center of Henan Province for Cancer Chemoprevention, Zhengzhou University, Zhengzhou 450001, Henan, China; 3 State Key Laboratory of Esophageal Cancer Prevention and Treatment, Zhengzhou University, Zhengzhou 450001, Henan, China

**Keywords:** STAT3, immune infiltration, drug response, bioinformatics

## Abstract

Signal transducer and activator of transcription 3 (STAT3) is a transcription factor and regulates tumorigenesis. However, the functions of STAT3 in immune and drug response in cancer remain elusive. Hence, we aim to reveal the impact of STAT3 in immune infiltration and drug response comprehensively by bioinformatics analysis. The expression of STAT3 and its relationship with tumor stage were explored by Tumor Immune Estimation Resource (TIMER), Human Protein Altas (HPA), and UALCAN databases. The correlations between STAT3 and immune infiltration, gene markers of immune cells were analyzed by TIMER. Moreover, the association between STAT3 and drug response was evaluated by the Cancer Cell Line Encyclopedia (CCLE) and Cancer Therapeutics Response Portal (CTRP). The results suggested that the mRNA transcriptional level of STAT3 was lower in tumors than normal tissues and mostly unrelated to tumor stage. Besides, the protein expression of STAT3 decreased in colorectal and renal cancer compared with normal tissues. Importantly, STAT3 was correlated with immune infiltration and particularly regulated tumor-associated macrophage (TAM), M2 macrophage, T-helper 1 (Th1), follicular helper T (Treg), and exhausted T-cells. Remarkably, STAT3 was closely correlated with the response to specified inhibitors and natural compounds in cancer. Furthermore, the association between STAT3 and drug response was highly cell line type dependent. Significantly, the study provides thorough insight that STAT3 is associated with immunosuppression, as well as drug response in clinical treatment.

## 1. Introduction

Signal transducer and activator of transcription 3 (STAT3) is a transcription factor transmitting signals from numerous receptors. Besides, it can also regulate the expression of various genes contributing to tumorigenesis [1,2,3]. STAT3 is constitutively activated by cytokines and growth factors in a large percentage of human tumors. Cytokine interleukin 6 (IL-6) and growth factor vascular endothelial growth factor (VEGF) can activate the tyrosine phosphorylation cascade. And STAT3 tightly mediates diverse processes influencing tumor progression, including proliferation, apoptosis, angiogenesis, and immune response [4,5,6].

Apart from tumor cells, STAT3 is also implicated in many other non-malignant cells in the tumor microenvironment [7]. The tumor microenvironment is a network containing various cells like fibroblasts, epithelial cells, cytokines, chemokines, and infiltrating immune cells [8]. In addition, numerous studies have shown that the tumor immune microenvironment is complex, and the tumor-infiltrating immune cells play the leading role in the tumor microenvironment. Infiltrating immune cells include T-cells, B-cells, monocytes, macrophages, natural killer cells, and dendritic cells (DCs). Moreover, the infiltrating immune cells are tightly associated with the progression of cancer [9]. Furthermore, aberrant STAT3 activation is also observed in tumor-interacting immune cells; it can increase the production of diverse immunosuppressive factors and decrease the number of immune activation factors [10,11]. Hyper-activated STAT3 regulates the expression of interferon γ (IFNγ), interleukin 12 (IL-12), CD86, C-C motif chemokine ligand 5 (CCL5), and C-X-C motif chemokine ligand 10 (CXCL10) in the tumor microenvironment to promote immune evasion [12,13]. Moreover, it has been investigated that STAT3 activation can suppress the maturation of DCs, meaning that STAT3 regulates the antigen-presenting process in the immune system [14]. Additionally, Janus Kinase (JAK)/STAT3 signaling is involved in the differentiation of immature DCs under the stimulation of the tumor microenvironment [15]. Programmed cell death protein 1 (PD-1) and programmed cell death ligand 1 (PD-L1) are important immune checkpoint molecules, and blocking PD-1/PD-L1 reduces the tumor burden and indicate promising outcomes in many tumor types [16,17]. Interestingly, it has ben reprted that STAT3 can bind to PD-L1 promoter in cancer cells [18,19], then induce immunosuppression by regulating PD-1/PD-L1 expression [20,21]. Although STAT3 is considered to be crucial to regulate the immune response in the tumor microenvironment, few studies have systematically reported the impact of STAT3 in different immune cells. Therefore, a detailed investigation of the association between STAT3 and multiple kinds of infiltrating immune cells is needed.

Growing evidence points to the aberrant activity of STAT3 in cancer providing a potential therapeutic option [22]. It has been demonstrated that metformin inhibits angiogenesis by suppressing the JAK/STAT3 signaling pathway in esophageal squamous cell carcinoma [6], and aspirin exhibits anticancer effects by regulating STAT3 signaling in colorectal cancer [23]. Aside from that, some kinase inhibitors targeting JAK and Src kinases have shown antitumor activity by disrupting STAT3 activity [24]. Plenty of small molecule compounds have been approved in cancer prevention and therapy by inhibiting the activity of STAT3 [25]. Although the role of STAT3 in tumorigenesis needs to be elucidated, it is clear that STAT3 is an advantageous drug target for cancer treatment. However, the efficacy of specific inhibitors regulating STAT3 varies among tumor types because the percentage of activated STAT3 is individualized in cancer cells. Then, a detailed analysis is needed to reveal the correlation between STAT3 and compounds in different cancer types, and it may provide a better understanding of STAT3 in cancer therapy.

Owing to the recent advancement in bioinformatics, we aim to reveal the impact of STAT3 in tumor immune microenvironment and drug response comprehensively by bioinformatics analysis. Our results shed light on the potential role of STAT3 in mediating the immune response in the tumor microenvironment. Moreover, the study have also indicated the association between STAT3 expression and drug response in different cancer cell lines. Importantly, the data provide a new insight into the combination of immunotherapy and STAT3 related inhibitors in clinical cancer treatment.

## 2. Materials and Methods

### 2.1. TIMER Database

The database Tumor Immune Estimation Resource (TIMER, https://cistrome.shinyapps.io/timer/) includes more than 10,000 samples across 23 cancer types from The Cancer Genome Atlas (TCGA) [26]. The database was used to explore the mRNA transcriptional level of STAT3 in multiple cancer types. As a convenient web-based resource, TIMER can also make the relationship between gene expression and tumor purity in cancer visible [27]. The gene module on TIMER estimates the correlation between gene expression and tumor-infiltrating immune cells (TIICs) [28]. Based on TIMER, we analyzed the relationship between STAT3 expression and tumor purity in diverse cancer types. We also evaluated the association between STAT3 and the abundance of six TIICs in cancer, including B-cells, CD8^+^ T-cells, CD4^+^ T-cells, macrophages, neutrophils, and dendritic cells. Some candidate marker genes of certain immune cells were selected from previous studies [29,30], then the correlation between STAT3 and these genes was also analyzed by TIMER. STAT3 was used for the *x*-axis with gene symbols, and related marker genes were represented on the *y*-axis as gene symbols. The transcriptional level was displayed with log2 RSEM. The correlation was exhibited by the purity-corrected partial Spearman method (partial-cor).

### 2.2. UALCAN Database

The database UALCAN (http://ualcan.path.uab.edu/) was used to analyze the correlation between STAT3 mRNA transcriptional level and pathological stage in cancer. The pathological stage in cancer was divided into Stages 1, 2, 3, and 4. The platform UALCAN is a web-based tool to analyze gene expression data deeply using genomics data from TCGA and clinical data from 31 cancer types [31].

### 2.3. The Human Protein Altas Database

The protein expressions of STAT3 in human normal tissues and tumor tissues were validated via the Human Protein Altas (HPA, https://www.proteinatlas.org/). The antibody used in the database was HPA001671. The HPA database represents the protein expression in 44 major human tissues and some cancer tissues by immunohistochemistry [32]. All the images and annotations in the database are available for download. The analysis of the data for the immunohistochemical staining of STAT3 protein expression refers to a previous study [33]. Statistical analysis was performed using SPSS version 21.0, and the *p*-value was determined using the Mann–Whitney U-test.

### 2.4. Drug Response

The mRNA profiles for 823 cancer cell lines were downloaded from the Cancer Cell Line Encyclopedia (CCLE, https://portals.broadinstitute.org/ccle/data). The drug response was measured by the area under the curves (AUCs) for 545 compounds across cancer cell lines, and the datasets were obtained from the Cancer Therapeutics Response Portal (CTRP, https://ocg.cancer.gov/programs/ctd2/dataporta), including CTRPv2.0 2015 ctd2 Expanded Dataset, CTRPv2.1 2016 pub Nat Chem Biol 12 109, and CTRPv2.2 2015 pub Cancer Disc 5 1210. The compounds contain FDA-approved drugs, clinical candidates, and small molecule probes. Then, the correlation between drug sensitivity and gene expression level was evaluated by Pearson correlation coefficients [34,35]. In the study, we used the transcriptomic data from CCLE and drug response data from CTRP to generate the correlation analysis between STAT3 expression and drug response AUC for all 823 cancer cell lines by Pearson correlation. Besides, the correlation between STAT3 expression and each cancer cell line type with at least 30 cell line replicates was analyzed separately. The threshold was set at a *p*-value of less than 0.05. The data analysis was conducted in RStudio Version 1.2.5033.

## 3. Results

### 3.1. The mRNA Expression of STAT3 and Correlation to Pathological Stage in Cancer

**Figure 1:**
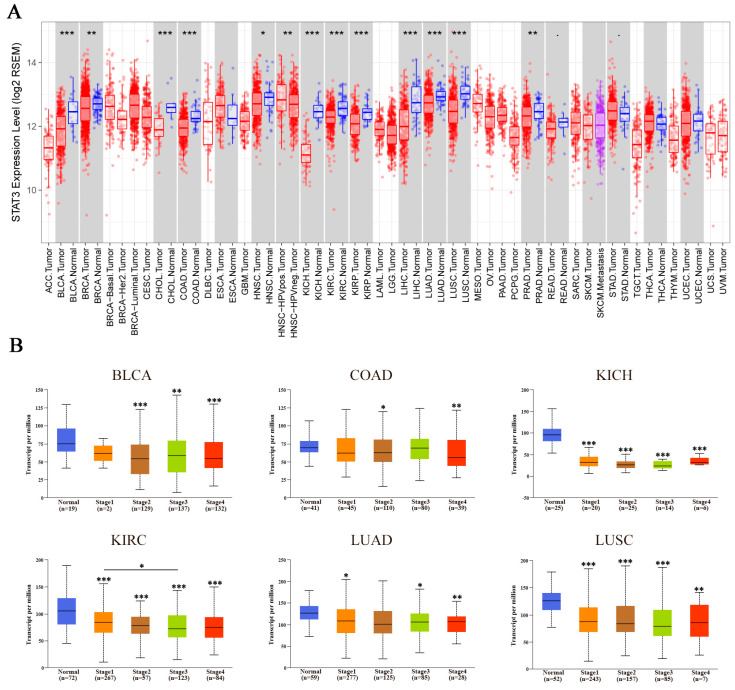
The expression levels of signal transducer and activator of transcription 3 (STAT3) along with the pathological stage in cancer. (**A**) The mRNA expression levels of STAT3 in different cancer types were explored by TIMER. (**B**) The correlation between the expression of STAT3 and the pathological stage in bladder urothelial carcinoma (BLCA), colon adenocarcinoma (COAD), kidney chromophobe (KICH), kidney renal clear cell carcinoma (KIRC), lung adenocarcinoma (LUAD), and lung squamous cell carcinoma (LUSC) was explored by UALCAN database. The first layer asterisk above the error bar represents a comparison to the normal group, and the secondary layers asterisk above a line represents the comparison between corresponding groups that were covered by the line. * *p* < 0.05, ** *p* < 0.01, *** *p* < 0.001.

The mRNA expression levels of STAT3 were explored by TIMER in many cancer types. Additionally, the results revealed that STAT3 mRNA expression levels were significantly lower in most cancer samples than their corresponding normal samples, including bladder urothelial carcinoma (BLCA), breast invasive carcinoma (BRCA), cholangiocarcinoma (CHOL), colon adenocarcinoma (COAD), head and neck squamous cell carcinoma (HNSC), kidney chromophobe (KICH), kidney renal clear cell carcinoma (KIRC), kidney renal papillary cell carcinoma (KIRP), liver hepatocellular carcinoma (LIHC), lung adenocarcinoma (LUAD), lung squamous cell carcinoma (LUSC), and prostate adenocarcinoma (PRAD). Besides, the data also showed that STAT3 expression was aberrantly higher in HPV-positive HNSC than HPV-negative HNSC (Figure 1A).

Subsequently, to have a better understanding of the relationship between STAT3 and clinicopathological features, we analyzed the STAT3 expression level in different pathological stages by UALCAN. The results showed that STAT3 expression was lower in different pathological stages of BLCA, COAD, KICH, KIRC, LUAD, and LUSC compared with normal tissues. Meanwhile, STAT3 in KIRC with Stage 3 was significantly lower than that with Stage 1. However, there was no significant difference in STAT3 expression level among Stages 1–4 in BLCA, COAD, KICH, LUAD, and LUSC (Figure 1B), suggesting that mRNA transcriptional level of STAT3 might not relate to pathological stages in these cancer types.

### 3.2. The Protein Expression of STAT3 in Cancer

**Figure 2:**
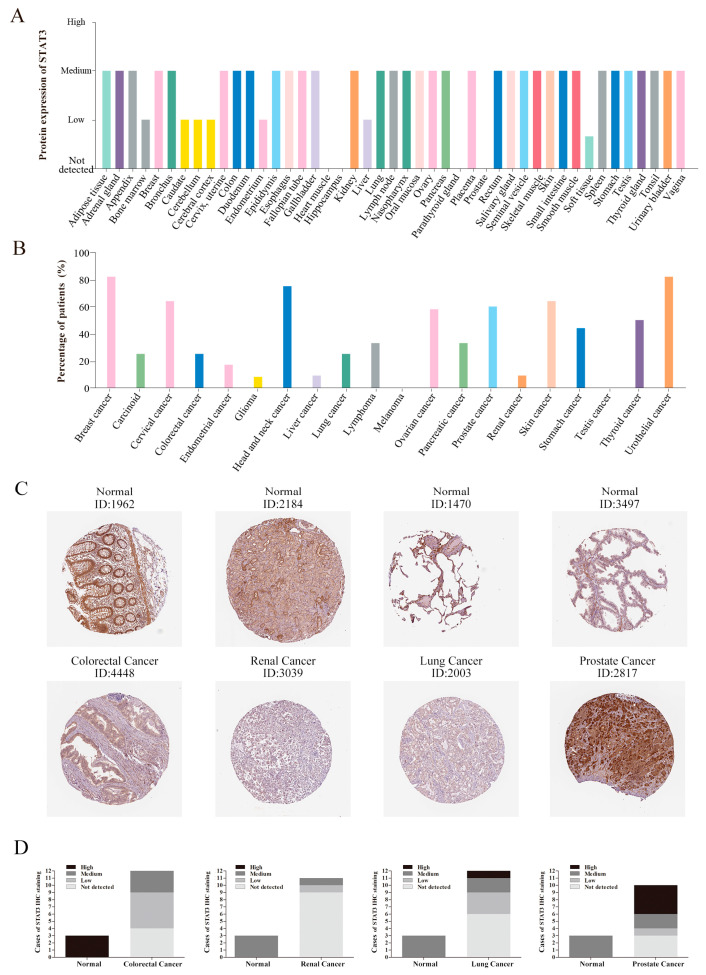
The protein expression of STAT3 in normal and cancer tissues. (**A**) The protein expression of STAT3 in human normal tissues. (**B**) The percentage of patients with high and medium STAT3 protein expression levels in different cancer types. The blank bar represents low or not detected protein expression. (**C**) Immunohistochemistry images of STAT3 in colorectal cancer, renal cancer, lung cancer, prostate cancer, and normal tissues detected in the HPA database. (**D**) Statistical analysis of STAT3 immunohistochemical staining data in normal tissues (*n* = 3) and colorectal cancer (*n* = 12, *p* = 0.007), renal cancer (*n* = 11, *p* = 0.006), lung cancer (*n* = 12, *p* = NS), prostate cancer (*n* = 10, *p* = NS) tissues.

Additionally, the protein expression level of STAT3 was found by using clinical specimens from the HPA database. The STAT3 protein expression data was shown for each of the 44 normal tissues (Figure 2A), and it revealed that most of the normal tissues presented medium staining. As for heart muscle, hippocampus, parathyroid gland, and prostate tissues, STAT3 was undetectable. Moreover, we also summarized the ratio of patients (maximum 12 patients) with the high and medium levels of STAT3 in 20 cancer tissues (Figure 2B). STAT3 protein expression levels might diverse in different cancer types. About 80% of patients with breast cancer, head and neck cancer, and urothelial cancer showed medium or high expression. Fewer patients with glioma, liver cancer, melanoma, and testis cancer could detect medium or high expression of STAT3. Compared with normal tissues, most kinds of cancer would display a lower protein expression of STAT3. Meanwhile, we also selected some typical immunohistochemistry images. The immunohistochemistry images revealed that STAT3 showed low staining in colorectal cancer, but high staining in normal colon tissue. Consistent with that, STAT3 protein expression was undetected in renal cancer and lung cancer, while the expression in normal tissues was medium. Hence, the protein expression of STAT3 in colorectal cancer, renal cancer, and lung cancer revealed lower levels compared with normal tissues, showing a similar result to mRNA transcriptional levels. However, the medium staining of STAT3 was found in normal prostate tissues and high staining in prostate cancer (Figure 2C), suggesting that the STAT3 protein expression was higher in prostate cancer than normal tissue. Although the statistical analysis (Figure 2D) showed that there was no significance for STAT3 staining in lung cancer and prostate cancer, which might be owing to the small sample size, the abnormal expression of STAT3 in cancer was obvious.

### 3.3. STAT3 Expression was Correlated with Immune Infiltration

**Figure 3:**
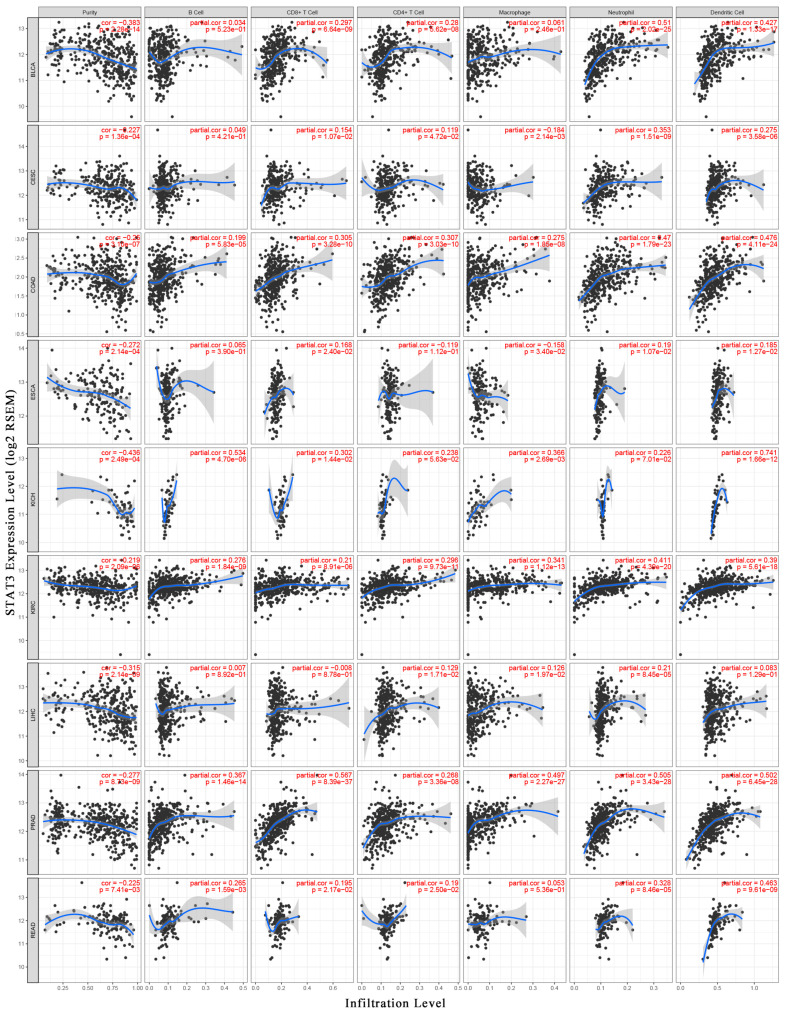
Correlation analysis between STAT3 and immune infiltration level. The correlations between STAT3 and tumor purity, STAT3 and infiltration levels of six TIICs (B-cells, CD4^+^ T-cells, CD8^+^ T-cells, macrophages, neutrophils, and dendritic cells) were analyzed respectively by TIMER in BLCA, cervical squamous cell carcinoma and endocervical adenocarcinoma (CESC), COAD, esophageal carcinoma (ESCA), KICH, KIRC, liver hepatocellular carcinoma (LIHC), prostate adenocarcinoma (PRAD), and rectum adenocarcinoma (READ).

To have a better understanding of STAT3 and the tumor immune microenvironment, we analyzed the association between STAT3 mRNA expression level and tumor purity using the TIMER database. Tumor purity means the percentage of tumor cells in the tumor tissue. Analysis of TIMER databases showed that STAT3 expression level was negatively related to tumor purity in nine cancer types significantly, including BLCA, cervical squamous cell carcinoma and endocervical adenocarcinoma (CESC), COAD, esophageal carcinoma (ESCA), KICH, KIRC, LIHC, PRAD, and rectum adenocarcinoma (READ). The results suggested that STAT3 expression level was inversely correlated with tumor purity, indicating that STAT3 was highly correlated with non-tumor cells, especially in BLCA (*r* = −0.383), KICH (*r* = −0.436), and LIHC (*r* = −0.315) (Figure 3).

Thereafter, the association between STAT3 expression level and abundance of TIICs was assessed. We observed that STAT3 expression had positive correlations with all six TIICs in KIRC and PRAD significantly. Interestingly, STAT3 was significantly correlated with infiltrating levels of B-cells in 4 cancer types (KICH, KIRC, PRAD, and READ), CD8^+^ T-cells in 5 cancer types (BLCA, COAD, KICH, KIRC, and PRAD), CD4^+^ T-cells in 4 cancer types (BLCA, COAD, KIRC, and PRAD), macrophage in 4 cancer types (COAD, KICH, KIRC, and PRAD), neutrophils in 7 cancer types (BLCA, CESC, COAD, KIRC, LIHC, PRAD, and READ), and DCs in 7 cancer types (BLCA, CESC, COAD, KICH, KIRC, PRAD, and READ). Additionally, the moderate correlations between STAT3 and neutrophils (*r* = 0.51), DCs (*r* = 0.427) were identified, respectively, in BLCA. Similarly, the moderate or strong correlations between STAT3 and infiltrating levels were shown, involving neutrophils (*r* = 0.47) and DC (*r* = 0.476) in COAD; B-cell (*r* = 0.534) and DC (*r* = 0.742) in KICH; neutrophils (*r* = 0.411) in KIRC; CD8^+^ T-cells (*r* = 0.567), macrophages (*r* = 0.497), neutrophils (*r* = 0.505) and DCs (*r* = 0.502) in PRAD; DCs (*r* = 0.463) in READ (Figure 3).

The results implied that STAT3 was associated with immune infiltration in BLCA, CESC, COAD, ESCA, KICH, KIRC, LIHC, PRAD, and READ. Although the correlation between STAT3 and abundance of TIICs was verified in different cancer types, STAT3 was particularly correlated with the infiltrating level of neutrophil and DC.

### 3.4. Correlation Analysis between STAT3 Expression and Immune Marker Genes

The correlation analysis between STAT3 and marker genes of certain immune cells was carried out by TIMER. The immune cells included CD8^+^ T-cells, monocytes, TAM, M1 macrophages, M2 macrophages, neutrophils, DCs, as well as various subsets of T-cells, to be specific, T-cells (general), T-helper 1 (Th1) cells, regulatory T (Tregs) cells, follicular helper T (Tfh) cells, and exhausted T-cells. Considering the correlation between STAT3 and tumor purity, STAT3 and abundance of TIICs, we focused on BLCA, KICH, and PRAD to study the correlation between STAT3 and immune cells. The results showed that STAT3 was positively correlated with most gene markers of immune cells in BLCA, KICH, and PRAD. Specifically, STAT3 showed significant correlation with CD^+^8 T-cell marker genes CD8A and CD8B; monocyte marker genes CD86 and CD115; TAM marker genes CCL2, CD68, and IL10; M1 macrophage marker genes inducible nitric oxide synthase (INOS), interferon regulator factor 5 (IRF5), and cytochrome c oxidase subunit II (COX2); M2 macrophage marker genes CD163, V-set and immunoglobulin domain containing 4 (VSIG4), and membrane-spanning 4-domains A4A (MS4A4A) (Table S1). The data also showed that the correlation between STAT3 and immune infiltration levels was higher in KICH than in BLCA and PRAD.

**Figure 4:**
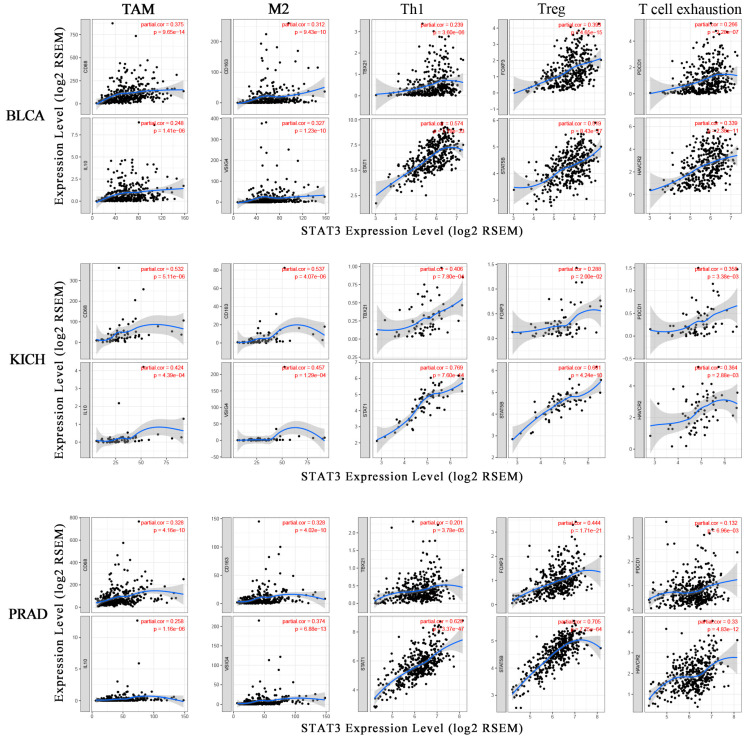
The correlation between STAT3 and marker genes of immune cells. The scatter plots showed the correlation between the STAT3 expression level and the gene markers of TAM (CD86, IL10); M2 macrophages (CD163, VSIG4); Th1 (TBX21, STAT1); Treg (FOXP3, STAT5B); T-cell exhaustion (PDCD1, HAVCR2) expression levels in BLCA, KICH, and PRAD.

At the same time, TAM markers CD68 and IL10, M2 macrophage markers CD163 and VSIG4, were closely correlated with STAT3 expression (Figure 4). TAMs are involved in various mechanisms of tumor biology, including tumor immune escape [36]. Apart from that, STAT3 was also associated with neutrophils infiltration in BLCA, KICH, and PRAD. Neutrophils marker genes C-C motif chemokine receptor 7 (CCR7) and CD11b, were favorably linked to STAT3 in cancer, except for CCR7 showing a negative correlation with STAT3 in BLCA (Table S1). Consistently, STAT3 positively related to DC infiltration in cancer. The expression of DC marker genes, like major histocompatibility complex, class II, DP beta 1 (HLA-DPB1), major histocompatibility complex, class II, DQ beta 1 (HLA-DQB1), major histocompatibility complex, class II, DR alpha (HLA-DRA), major histocompatibility complex, class II, DP alpha 1 (HLA-DPA1), BDCA-1, BDCA-4, and CD11c were strongly correlated with STAT3 expression (Table S1). These results further exposed that STAT3 might correlate with macrophages and DCs.

In addition, we also revealed the significant positive correlation between STAT3 and marker genes of different subsets of T-cells, including T-cell (general) marker genes CD3E and CD2; Th1 marker genes, T-bet, signal transducer and activator of transcription 4 (STAT4), signal transducer and activator of transcription 1 (STAT1), and tumor necrosis factor alpha-like (TNF-α); Treg marker genes forkhead box P3 (FOXP3) and signal transducer and activator of transcription 5B (STAT5B); Tfh marker genes BCL6 and interleukin 21 (IL21); exhausted T-cell marker genes PD-1, cytotoxic T-lymphocyte associated protein 4 (CTLA4), and TIM-3 (Table S1). Furthermore, T-bet (TBX21) and STAT1, as crucial transcription factors regulating Th1, were positively correlated with STAT3 (Figure 4). FOXP3 and STAT5B, with vital roles in immune suppression, had significant positive correlations with STAT3 expression (Figure 4). As the potential targets for immunotherapy, PD-1 (PDCD1) and TIM-3 (HAVCR2) expression levels showed a strong relationship with STAT3 (Figure 4). These findings suggested that STAT3 was closely related to immune escape in the tumor microenvironment.

### 3.5. STAT3 Expression and Drug Response

**Figure 5:**
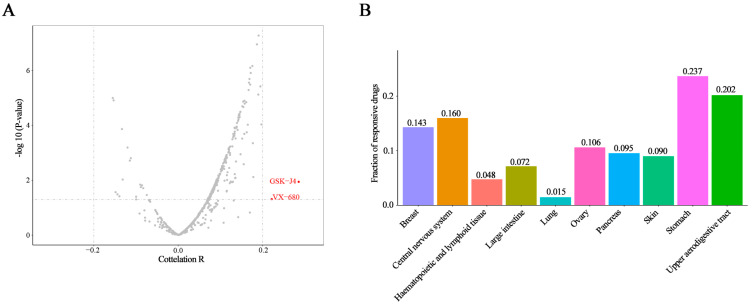
STAT3 expression and drug response correlation. (**A**) The volcano plots showed the correlation coefficient (*x*-axis) and −log10 *p*-value (*y*-axis) between STAT3 expression and drug response in 823 cancer cell lines and 545 drugs. The red dots were the drugs with a correlation coefficient greater than 0.2 and a *p*-value less than 0.05. (**B**) The histogram showed the ratio of drugs with a correlation coefficient greater than 0.3 and a *p*-value less than 0.05 in 10 cancer cell line types.

A combination of immunotherapy and small molecule inhibitors was an effective choice and targeting STAT3 directly or indirectly was also the potential approach for cancer therapy. So, we evaluated the association between STAT3 expression and the response to 545 drugs in 823 cancer cell lines by CCLE and CTRP. Additionally, the results showed that only two drugs, VX-680 and GSK-J4, had a correlation coefficient greater than 0.2 (both were positively correlated, Figure 5A). The positive correlation means that higher expression of this gene was associated with resistance. A negative correlation suggests that higher expression of the gene is correlated with a better response (smaller response AUC value) [37]. VX-680 is the inhibitor of pan-aurora kinases, and GSK-J4 is a histone demethylase inhibitor. Furthermore, both VX-680 and GSK-J4 have shown the anticancer effect [38,39]. In order to analyze the results systematically, the cancer cell lines were divided into 10 types according to different primary sites in the CCLE database, containing breast, central nervous system, hematopoietic and lymphoid tissue, large intestine, lung, ovary, pancreas, skin, stomach, and upper aerodigestive tract. Interestingly, ratios of drugs associated with STAT3 expression were quite diverse among these cancer cell lines and highly primary-site-dependent (Figure 5B). More than 20% of drugs showing a correlation coefficient higher than 0.3 were collected in the stomach cancer cell lines, while only 1.5% of drugs exhibited an association with STAT3 expression in lung cancer (Figure 5B). The volcano plots showed that more than one compound displayed a correlation coefficient higher than 0.3 with STAT3 expression in the 10 cancer cell lines types, respectively (Figure S1). Except for lung cancer cell lines, there were more than 20 compounds with correlation coefficients greater than 0.3. Then, we summarized the top 10 compounds whose drug response were significantly related to STAT3 expression in 10 cancer cell line types separately (Table 1).

In breast cancer, abiraterone, targeting CYP17A1, was negatively correlated with STAT3 (*r* = −0.969) to the greatest extent compared with other compounds. In other words, STAT3 expression was correlated with sensitivity to abiraterone in breast cancer cell lines. Abiraterone not only was approved by the FDA for the treatment of prostate cancer but also displayed the potential benefit for breast cancer [40,41]. Additionally, the probe bafilomycin A1, the target of ATP6V0A1, showed the highest correlation coefficient (*r* = −0.999) in skin cancer. Bafilomycin A1 is a promising candidate drug targeting growth, apoptosis, and metastasis in cancer cell lines [42,43], and it can also reduce cell viability by inducing autophagy in melanoma [44]. The data showed that STAT3 expression was correlated with sensitivity to bafilomycin A1 in skin cancer cell lines, and STAT3 might play a role in autophagy in skin cancer. In upper aerodigestive tract cancer cell lines, JW-55, which was the probe targeting TNKS, had a positive correlation coefficient of 0.998. JW-55 was the selective Wnt/β-catenin signaling pathway inhibitor [45], and STAT3 was also approved to regulate Wnt/β-catenin signaling in the development of cancer [46]. The strong correlation between STAT3 and JW-55 was likely explained in this way. While JW-55 showed a negative correlation (−0.655) with STAT3 expression in large intestine cancer, the difference might be owing to the fact that STAT3 expression was varied among different cancer cell lines. Among these, we also found the drug cerulenin targeting FASN in stomach cancer was correlated with STAT3 positively (*r* = 0.555). As an inhibitor of fatty acid synthase, cerulenin could affect the proliferation and metastasis of cancer cells [47]. Hence, these results suggested that STAT3 might regulate lipid homeostasis in stomach cancer. Several compounds were targeting the PI3K/AKT pathway, such as rigosertib, MK-2206, AZD6482, TGX-221, NVP-BEZ235, and targeting the JAK/STAT pathway, like ruxolitinib and AZD1480, which were significantly correlated to STAT3 expression in cancer cell lines. Consistent with our data, STAT3 was involved in the PI3K signaling pathway in cancer [48].

To further investigate the potential mechanism of phytochemical approaches to cancer treatment, we summarized the correlation between transcriptional expression level of STAT3 and sensitivity to natural compounds in cancer cell lines by CCLE and CTRP databases (Table 2). We found that STAT3 expression was significantly correlated with natural product betulinic acid, phloretin, nakiterpiosin, and cucurbitacin I. Among them, betulinic acid, which had the negative correlation with STAT3 expression in the large intestine cancer, possessed an antitumor property in breast cancer [49,50].

Besides, some reports revealed that betulinic acid could regulate STAT3 in many cancer types [51,52]. In central nervous system cancer cell lines, compounds nakiterpiosin and cucurbitacin I had a positive correlation coefficient greater than 0.3 (higher expression of STAT3 related to resistance). Apart from these, the response of phloretin was associated with STAT3 expression in upper aerodigestive tract cancer. Besides, the anti-inflammatory and anticancer effects of phloretin have been evaluated in many studies [53]. In total, the results turned out that some natural compounds might regulate STAT3 in cancer.

## 4. Discussion

Bioinformatics has been rapidly applied to cancer research in recent years. Using some powerful bioinformatics tools like software packages and databases, profiles of gene expression in cancer can be analyzed. With the aid of some statistical methods, bioinformatics can provide a platform to discover the underlying molecular mechanisms in cancer biology and development [54]. Here, using the microarray database, we explored the mRNA expression of STAT3 and found that STAT3 was significantly downregulated in various cancer samples compared with normal samples. And the protein expression of STAT3 was significantly decreased in colorectal cancer and renal cancer compared with normal tissues. Although there are some studies showing that STAT3 may be a tumor suppressor [55,56], STAT3 has been identified as an oncogene contributing progression in cancer [57,58]. Actually, STAT3 pathway was constitutively activated in cancer, and only after activation via phosphorylation, STAT3 was translocated to the nucleus and act as the oncogenic-associated protein [59]. The expression level of p-STAT3 could reflect the activity in cancer progression better than total STAT3 protein expression. Besides, the maximum sample size of tumor patients was 12 in the HPA dataset, which made the protein expression of STAT3 in cancer less objective and needed further investigation and verification. Furthermore, our data showed that STAT3 expression was not related to the pathological stage except KIRC. Consistent with that, it has been reported that STAT3 is not associated with pathological stages in colorectal cancer [60].

Apart from the known role of STAT3 in promoting cancer cell proliferation, invasion, and angiogenesis, STAT3 also showed the crucial role in immunosuppression [61]. In this study, we demonstrated that STAT3 expression was closely correlated with immune infiltration in cancer. The tumor tissues are composed of tumor cells and non-tumor cells, and tumor purity is the proportion of tumor cells in the mixture. Apart from immune cells, stromal, endothelial, and epithelial cells are also components of solid tumor tissues [62]. Considering STAT3 was closely related to immune cells in BLCA, KICH, and PRAD, so that we chose the three cancer types to investigate the correlation between STAT3 expression and the marker genes of immune cells. We also detected the correlations between STAT3 and TAM markers, M2 macrophage markers. CD68 is a pan-macrophage marker that points to both M1- and M2-activated TAM, while CD163 only refers to M2 macrophage-related antigen [63]. STAT3 correlated with CD68 and CD163, meaning that STAT3 might correlate with M2 macrophage to regulate cancer development. Consistent with that, some studies previously reported that M2 macrophage secreted IL-8 via the STAT3/MALAT1 pathway to promote prostate tumorigenesis [64], and ERK/STAT3 axis could drive breast cancer progressions by stimulating M2 macrophage [65]. Besides, our results also revealed that STAT3 was also correlated with gene markers of Tregs. Tregs, which were enriched in the tumor microenvironment in a high degree, were well known for the roles in immune suppression in tumors [66]. Following that, impeding the expression of STAT3 by some small molecules attributed to decreasing the infiltration of Tregs the tumor microenvironment [67]. FOXP3, the lineage-specification factor, is essential for maintaining the suppressor function of Tregs. It can also act as a co-transcription factor of STAT3 in Tregs [68]. Furthermore, the data showed that STAT3 might induce T-cell exhaustion. TIM-3, a vital surface protein on exhausted T-cells [69], could mediate immune exhaustion in the tumor microenvironment. As a new immune checkpoint molecule, TIM-3 promotes tumorigenesis through the NF-κB/IL-6/STAT3 axis [70]. PD-1 is another immune checkpoint molecule, and cancer cells utilize PD-1/PD-L1 to evade T-cell-mediated tumor specific immunity in the tumor microenvironment [71]. Moreover, the direct link between STAT3 and PD-1 in tumor has been previously described [20,72]. Taken together, STAT3 might correlate with M2 macrophage, Tregs, and T-cell exhaustion, regulating immune suppression in the tumor microenvironment.

More and more studies have shown that combination of immunotherapy and small molecule inhibitors is more effective than immunotherapy alone. Apart from the role of STAT3 in immune suppression, this study also demonstrates the significant association between STAT3 expression and drug response. The data was interesting that the significant association between STAT3 expression and VX-680, GSK-J4 treatment response in 823 cell lines. VX-680 might mediate cell death by acting on P53/Bax/caspase-3 dependent pathway in cholangiocarcinoma cell [73]. Additionally, oxaliplatin-induced NOTCH signaling could be interrupted by GSK-J4 treatment in colorectal cancer [74], while there was no evidence showing that both VX-680 and GSK-J4 regulated STAT3 in the progression of tumors. Significantly, our study provided some clues for the mechanism research of the two drugs in antitumor therapy. Recently, many studies have shown that lipogenesis is a crucial characteristic in the tumor microenvironment. And targeting signaling cascades about lipid homeostasis is a promising way in cancer therapy [75]. Interestingly, we found that the expression of STAT3 was closely correlated with the response of fatty acid synthase inhibitor. Based on this, understanding STAT3 related mechanisms, like regulating lipogenesis, was essential to understand cancer initiation and progression. Over these years, natural compounds extracting from traditional Chinese herbs have been investigated as an advantageous approach to cancer therapy. Meanwhile, our team formerly found that natural compounds curcumin could play an important role in angiogenesis by regulating STAT3 [76]. In this current study, we also found betulinic acid responded to STAT3 expression in large intestine cancer. Consistent with our results, Dan Su et al. identified that betulinic acid showed potent therapeutic and antitumor effects through the NF-*κ*B and STAT3 pathways in HT-29 colorectal cancer cell [77]. Additionally, betulinic acid could downregulate STAT3 activation through SHP-1 in myeloma cells [78]. Overall, the data revealed that betulinic acid regulated STAT3 in cancer, suggesting the potential role of betulinic acid as a chemopreventive compound targeting STAT3. Cucurbitacins contained twelve categories [79], and showed the anticancer effect by different mechanisms, including JAK/STAT3, MAPK, PI3K/AKT, and NF-κB signaling pathways [80]. Our results showed that cucurbitacin I had a positive correlation with STAT3 in the central nervous system, meaning that higher expression of STAT3 was correlated with worse response. However, cucurbitacin I had been proved as a potential inhibitor of STAT3 [81,82]. The converse might be explained by the tumor heterogeneity and the drug response was highly cancer-type-dependent. Overall, these data laid the foundation for the study that betulinic acid and cucurbitacin I might regulate STAT3 in cancer.

To be honest, there were some limitations in this study. First, the maximum sample size of cancer patients was 12 and normal was 3 in the HPA dataset, which made the protein expression of STAT3 in cancer less objective. Moreover, the sample size of KICH was relatively small and the correlation with immune infiltration in KICH was less reliable compared with BLCA and PRAD. Therefore, succeeding studies are still needed to address these problems. Although there were limitations in our study, some of the results in our study were pretty meaningful and could offer directions for future study. For instance, we comprehensively analyzed the association between STAT3 and TIICs and our results showed that the correlation between STAT3 and M2 macrophage, Tregs, and T-cell exhaustion was higher compared with other immune cell types. As we know, M2 macrophages, Tregs, and T-cell exhaustion play an important role in immune suppression. Based on the above findings, we made the conclusion that STAT3 was particularly correlated with immune suppression in the tumor microenvironment. Furthermore, we systematically investigated the association between STAT3 expression and drug response in various cancer cell lines. Interestingly, our results revealed that VX-680 and GSK-J4 showed a positive response to STAT3 in cancer. Besides, the response of FASN inhibitor was closely correlated with STAT3, which provided insight into STAT3 might regulate lipid homeostasis. In addition, our results also revealed that some natural compounds might mediate STAT3 in cancer.

## 5. Conclusions

In summary, our study suggests that STAT3 is related to immune cell infiltration and closely associated with immune suppression. Additionally, STAT3 expression is tightly correlated with specified inhibitors and natural compounds treatment response in cancer. Therefore, STAT3 may play an important role in immune infiltration and cancer clinical therapy.

## Figures and Tables

**Table 1:** The correlation between STAT3 expression and drug response (Top 10).

Primary Site	Compound	Correlation *r*	*p*-Value	Status	Gene Symbol of Protein Target	Target or Activity of Compound
Breast						
	abiraterone	**−0.969**	0.031	FDA	CYP17A1	inhibitor of 17 alpha-hydroxylase and C17, 20 lyase
	bafilomycin A1	**0.762**	0.046	probe	ATP6V0A1	inhibitor of the vacuolar-type H+-ATPase
	BRD-K84807411	**−0.717**	0.006	GE-active		product of diversity-oriented synthesis
	BEC	**−0.705**	0.007	probe	ARG1; ARG2	inhibitor of arginase I and II
	BRD-K30019337	**−0.595**	0.032	GE-active		product of diversity-oriented synthesis
	fulvestrant	**0.590**	0.043	FDA	ESR1; GPER1	antagonist of the estrogen receptor
	MLN2238	**−0.524**	0.001	clinical	PSMB5	inhibitor of 20S proteasome at the chymotrypsin-like proteolytic (β−5) site
	BRD-K42260513	**−0.524**	0.026	probe	EZH2	inhibitor of enhancer of zeste polycomb repressive complex 2 subunit
	SB-525334	**0.523**	0.001	probe	TGFBR1	inhibitor of the transforming growth factor beta type 1 receptor
	sildenafil	**0.474**	0.003	FDA	PDE5	inhibitor of phosphodiesterase type 5
Central nervous system						
	docetaxel	**0.654**	0.006	FDA		inhibitor of microtubule depolymerization
	myriocin	**−0.612**	0.012	probe	SPTLC1; SPTLC2; SPTLC3	inhibitor of serine-palmitoyl-transferase
	GSK461364	**0.604**	0.000	clinical	PLK1	inhibitor of polo-like kinase 1 (PLK1)
	sitagliptin	**0.591**	0.016	FDA	DPP4	inhibitor of dipetidyl peptidase 4
	AT13387	**0.564**	0.006	clinical	HSP90	inhibitor of HSP90
	vincristine	**0.549**	0.000	FDA		inhibitor of microtubule assembly
	rigosertib	**0.538**	0.000	clinical	PIK3; PLK1	inhibitor of polo-like kinase 1; inhibitor of PI3K catalytic subunits α and β
	triazolothiadiazine	**0.529**	0.000	probe	PDE4A; PDE4B; PDE4D	inhibitor of phosphdiesterase 4A/B/D
	BRD-K70511574	**0.522**	0.000	probe	PLK1	inhibitor of polo-like kinase 1 (PLK1)
	CHM-1	**0.522**	0.000	probe		inhibitor of tubulin polymerization
Hematopoietic and lymphoid tissue						
	VX-680	**0.618**	0.024	clinical	AURKA; AURKB; AURKC	inhibitor of aurora kinases
	staurosporine	**0.505**	0.002	probe		inhibitor of multiple kinases
	YM-155	**0.430**	0.000	clinical	BIRC5	inhibitor of survivin expression
	BRD-K79669418	**0.399**	0.000	probe	MDM2; MDM4	inhibitor of MDM4-p53 interaction
	ruxolitinib	**−0.389**	0.000	FDA	JAK1; JAK2	inhibitor of Janus kinases 1 and 2
	ML239	**−0.360**	0.000	probe		inhibitor of breast cancer stem cell proliferation
	MK-2206	**0.356**	0.000	clinical	AKT1	inhibitor of AKT1
	AZD6482	**0.351**	0.000	clinical	PIK3CB; PIK3CD	inhibitor of PI3K catalytic subunits beta and delta
	sitagliptin	**0.349**	0.043	FDA	DPP4	inhibitor of dipetidyl peptidase-4
	AZD1480	**−0.348**	0.011	clinical	JAK1; JAK2	inhibitor of Janus kinases 1 and 2
Large intestine						
	JW-55	**−0.655**	0.006	probe	TNKS	inhibitor of tankyrase
	O-6-benzylguanine	**−0.569**	0.017	clinical	MGMT	inhibitor of O(6)-alkylguanine DNA alkyltransferases
	ML210	**−0.488**	0.001	probe		selectively kills engineered cells expressing mutant HRAS
	ML162	**−0.446**	0.002	probe		selectively kills engineered cells expressing mutant HRAS
	PRL-3 inhibitor I	**−0.437**	0.003	probe	PTP4A3	inhibitor of phosphatase of regenerating liver-3 (PRL3)
	imatinib	**−0.432**	0.003	FDA	ABL1; BCR; KIT	inhibitor of BCR-ABL1 and c-KIT
	1S,3R-RSL-3	**−0.421**	0.003	probe	GPX4	synthetic lethal with HRAS in engineered cells; inhibitor of GPX4
	betulinic acid	**−0.396**	0.008	probe		natural product; inhibitor of specificity protein 1 transcription factor in cells
	KH-CB19	**−0.391**	0.024	probe	CLK1; CLK4	inhibitor of CDC2-like kinases 1 and 4
	palmostatin B	**−0.386**	0.026	probe	LYPLA1	inhibitor of acyl-protein thioesterase 1
Lung						
	GSK-J4	**0.608**	0.047	probe	KDM6A; KDM6B	inhibitor of lysine-specific demethylases
	BRD-K49290616	**−0.376**	0.011	GE-active		product of diversity-oriented synthesis
	TGX-221	**−0.325**	0.000	probe	PIK3CB	inhibitor of PI3K catalytic subunit beta
	BRD-K96431673	**−0.302**	0.046	GE-active		product of diversity-oriented synthesis
Ovary						
	istradefylline	**0.586**	0.028	clinical	ADORA2A	antagonist of the adenosine A2A receptor
	BRD-K03536150	**0.507**	0.001	probe	BAX	activator of BAX
	LY-2183240	**0.448**	0.005	probe	FAAH	inhibitor of fatty acid amide hydrolase; inhibitor of anandamide uptake
	fumonisin B1	**−0.447**	0.004	probe	CERS1; CERS2; CERS3; CERS4; CERS5	inhibitor of ceramide synthase
	manumycin A	**0.437**	0.006	probe	FNTA; FNTB	inhibitor of RAS farnesyltransferase
	ML311	**0.431**	0.006	probe	MCL1; Bim	inhibitor of the Mcl-1/Bim interaction
	methotrexate	**0.421**	0.010	FDA	DHFR	inhibitor of dihydrofolate reductase
	SCH-79797	**0.411**	0.009	FDA	DHFR	inhibitor of dihydrofolate reductase
	BRD-K94991378	**0.378**	0.019	probe	F2R	antagonist of proteinase-activated receptor 1 (PAR1)
	ibrutinib	**−0.373**	0.047	clinical	BTK	inhibitor of Bruton’s tyrosine kinase
Pancreas						
	CIL70	**−0.500**	0.034	probe		screening hit
	RITA	**0.401**	0.011	probe	MDM2; TP53	inhibitor of p53-MDM2 interaction
	sotrastaurin	**0.400**	0.028	clinical	PKC	inhibitor of protein kinase C
	NVP-BEZ235	**0.386**	0.032	clinical	MTOR;PIK3CA; PIK3CB; PIK3CD	inhibitor of PI3K and mTOR kinase activity
	purmorphamine	**0.384**	0.016	probe	SMO	activator of smoothened receptor
	dexamethasone	**0.365**	0.022	FDA	NR3C1	agonist of glucocorticoid receptor
	doxorubicin	**−0.363**	0.023	FDA	TOP2A	inhibitor of topoisomerase II
	BRD8899	**0.358**	0.0275	probe	STK33	inhibitor of serine/threonine kinasase STK33
	cabozantinib	**−0.355**	0.0290	FDA	FLT3; KDR; MET; RET	inhibitor of c-MET, VEGFR2/3, and RET
	PF-750	**0.354**	0.0271	probe	FAAH	inhibitor of fatty acid amide hydrolase
Skin						
	bafilomycin A1	**−0.999**	0.023	probe	ATP6V0A1	inhibitor of the vacuolar-type H+-ATPase
	BRD-K09344309	**0.662**	0.01	probe		screening hit
	tigecycline	**−0.515**	0.029	FDA		analog of tetracycline
	sotrastaurin	**0.475**	0.001	clinical	PKC	inhibitor of protein kinase C
	HBX-41108	**−0.432**	0.003	probe	USP7	inhibitor of the deubiquitinase activity of USP7
	itraconazole	**−0.414**	0.003	FDA		anti-fungal agent; inhibitor of hedgehog signaling pathway
	1S,3R-RSL-3	**−0.409**	0.002	probe	GPX4	synthetic lethal with HRAS in engineered cells; inhibitor of GPX4
	bleomycin A2	**−0.406**	0.005	FDA		inducer of DNA damage
	indisulam	**−0.376**	0.008	clinical	CA9	inhibitor of carbonic anhydrase isoform IX
	ML162	**−0.371**	0.007	probe		selectively kills engineered cells expressing mutant HRAS
Stomach						
	KH-CB19	**0.567**	0.014	probe	CLK1; CLK4	inhibitor of CDC2-like kinases 1 and 4
	cerulenin	**0.555**	0.001	probe	FASN; HMGCS1	inhibitor of fatty acid synthase; inhibitor of HMG-CoA synthase
	obatoclax	**0.554**	0.001	clinical	BCL2; BCL2L1; MCL1	inhibitor of MCL1, BCL2, and BCL-xL
	fingolimod	**0.551**	0.001	FDA	S1PR1	inhibitor of sphingosine 1-phosphate receptor
	quizartinib	**0.539**	0.002	clinical	FLT3	inhibtor of VEGFR3
	ouabain	**0.538**	0.001	probe	ATP1A1; ATP1A2; ATP1A3; ATP1B1	cardiac glycoside; inhibitor of the Na+/K+-ATPase
	B02	**0.511**	0.003	probe	RAD51	inhibitor of RAD51
	darinaparsin	**−0.500**	0.029	clinical		inducer of ROS; inhibitor of microtubule assembly
	vorapaxar	**0.492**	0.004	clinical	F2R	antagonist of proteinase-activated receptor 1 (PAR1)
	cyclophosphamide	**0.473**	0.047	FDA		DNA alkylator
Upper aerodigestive tract						
	JW-55	**0.998**	0.007	probe	TNKS	inhibitor of tankyrase
	BRD-K29086754	**−0.735**	0.043	GE-active		product of diversity-oriented synthesis
	nutlin-3	**0.712**	0.015	clinical	MDM2	inhibitor of p53-MDM2 interaction
	BRD-K49290616	**−0.699**	0.000	GE-active		product of diversity-oriented synthesis
	BRD-K48334597	**−0.682**	0.017	GE-active		product of diversity-oriented synthesis
	CIL55A	**0.678**	0.021	probe		screening hit
	BRD-K34485477	**−0.618**	0.008	GE-active		product of diversity-oriented synthesis
	NSC48300	**0.591**	0.043	probe	TASP1	inhibitor of threonine endopeptidase taspase 1
	linifanib	**0.589**	0.001	clinical	FLT1; FLT3; KDR	inhibitor of VEGFRs
	tubastatin A	**−0.548**	0.001	probe	HDAC6	inhibitor of tubulin deacetylase activity of HDAC6

**Table 2:** The correlation between STAT3 expression and sensitivity to natural compounds.

Primary Site	Compound	Correlation *r*	*p*-Value	Status	Gene Symbol of Protein Target	Target or Activity of Compound
Central nervous system						
	nakiterpiosin	**0.381**	0.013	probe		natural product; inhibitor of microtubule assembly
	cucurbitacin I	**0.338**	0.031	probe		natural product; modulator of NFKB1 and STAT3 signaling
Large intestine						
	betulinic acid	**−0.396**	0.008	probe		natural product; inhibitor of specificity protein 1 transcription factor in cells
Upper aerodigestive tract						
	phloretin	**0.425**	0.022	probe	SLC5A1	natural product; inhibitor of glucose uptake

## Supplementary Materials

The following are available online at https://www.mdpi.com/2218-273X/10/6/834/s1, Figure S1: STAT3 expression and drug response correlation in 10 types of cancer cell lines, Table S1: Correlation analysis between STAT3 and gene markers of immune cells in TIMER.

## Abbreviations

AUC	area under the curve
BLCA	bladder urothelial carcinoma
BRCA	breast invasive carcinoma
CCL5	C-C motif chemokine ligand 5
CCLE	Cancer Cell Line Encyclopedia
CCR7	C-C motif chemokine receptor 7
CESC	cervical squamous cell carcinoma and endocervical adenocarcinoma
CHOL	cholangiocarcinoma
COAD	colon adenocarcinoma
COX2	cytochrome c oxidase subunit II
CTLA4	cytotoxic T-lymphocyte associated protein 4
CTRP	Cancer Therapeutics Response Portal
CXCL10	C-X-C motif chemokine ligand 10
DC	dendritic cell
ESCA	esophageal carcinoma
FOXP3	forkhead box P3
HLA-DPA1	major histocompatibility complex, class II, DP alpha 1
HLA-DPB1	major histocompatibility complex, class II, DP beta 1
HLA-DQB1	major histocompatibility complex, class II, DQ beta 1
HLA-DRA	major histocompatibility complex, class II, DR alpha
HNSC	head and neck squamous cell carcinoma
HPA	Human Protein Atlas
IFNγ	interferon γ
IL-12	interleukin 12
IL21	interleukin 21
IL-6	interleukin 6
INOS	inducible nitric oxide synthase
IRF5	interferon regulator factor 5
JAK	Janus kinaseKICH, kidney chromophobe
KIRC	kidney renal clear cell carcinoma
KIRP	kidney renal papillary cell carcinoma
LIHC	liver hepatocellular carcinoma
LUAD	lung adenocarcinoma
LUSC	lung squamous cell carcinoma
MS4A4A	membrane spanning 4-domains A4A
PD-1	programmed cell death protein 1
PD-L1	programmed cell death ligand 1
PRAD	prostate adenocarcinoma
READ	rectum adenocarcinoma
STAT1	signal transducer and activator of transcription 1
STAT3	Signal transducer and activator of transcription 3
STAT4	signal transducer and activator of transcription 4
STAT5B	signal transducer and activator of transcription 5B
TAM	tumor-associated macrophage
TCGA	The Cancer Genome Atlas
Th1	T-helper 1
TIICs	tumor-infiltrating immune cells
TIMER	Tumor Immune Estimation Resource
TNF-α	tumor necrosis factor alpha-like
Treg	follicular helper T
VEGF	vascular endothelial growth factor
VSIG4	V-set and immunoglobulin domain containing 4
