# Supplementary material for: Significance of STAT3 in Immune Infiltration and Drug Response in Cancer
Source: Biomolecules. 2020 May 29;10(6):834. doi: 10.3390/biom10060834 (PMC7355836; doi:10.3390/biom10060834)
Supplement: Supplementary file 1 [file biomolecules-10-00834-s001.pdf]

Table 1. Correlation analysis between STAT3 and gene markers of immune cells in TIMER.

Immune Cells	Gene Markers	BLCA		KICH		PRAD	
		Cor	*p*	Cor	*p*	Cor	*p*
**CD8⁺Tcell**	CD8A	**0.253**	***	**0.299**	*	**0.313**	***
	CD8B	**0.208**	***	**0.143**	0.25	**0.159**	**
Monocyte	CD86	**0.371**	***	**0.590**	***	**0.352**	***
	CD115 (CSF1R)	**0.335**	***	**0.515**	***	**0.407**	***
TAM	CCL2	**0.207**	***	**0.445**	***	**0.235**	***
	CD68	**0.375**	***	**0.532**	***	**0.314**	***
	IL10	**0.248**	***	**0.424**	***	**0.332**	***
M1 Macrophage	INOS (NOS2)	**0.291**	***	**0.236**	0.06	**0.350**	***
	IRF5	**-0.05**	0.30	**0.462**	***	**0.365**	***
	COX2 (PTGS2)	**0.233**	***	**0.176**	0.16	**0.375**	***
M2 Macrophage	CD163	**0.312**	***	**0.537**	***	**0.424**	***
	VSIG4	**0.327**	***	**0.457**	***	**0.362**	***
	MS4A4A	**0.239**	***	**0.55**	***	**0.305**	***
Neutrophils	CD11b (ITGAM)	**0.35**	***	**0.51**	***	**0.418**	***
	CCR7	**-0.32**	***	**0.433**	***	**0.286**	***
Dendritic cell	HLA-DPB1	**0.274**	***	**0.506**	***	**0.146**	**
	HLA-DQB1	**0.294**	***	**0.304**	*	**0.170**	***
	HLA-DRA	**0.374**	***	**0.531**	***	**0.357**	***
	HLA-DPA1	**0.347**	***	**0.489**	***	**0.330**	***
	BDCA-1 (CD1C)	**0.199**	***	**0.386**	**	**0.343**	***
	BDCA-4 (NRP1)	**0.371**	***	**0.476**	***	**0.413**	***
	CD11c	**0.302**	***	**0.435**	***	**0.327**	***
**Different types of T-cells**							
T cell (general)	CD3E	**0.263**	***	**0.374**	**	**0.204**	***
	CD2	**0.253**	***	**0.358**	**	**0.195**	***
Th1	T-bet (TBX21)	**0.239**	***	**0.406**	***	**0.201**	***
	STAT4	**0.437**	***	**0.371**	**	**0.255**	***
	STAT1	**0.574**	***	**0.769**	***	**0.629**	***
	TNF-α (TNF)	**0.288**	***	**0.488**	***	**0.202**	***
Treg	FOXP3	**0.393**	***	**0.288**	*	**0.444**	***
	STAT5B	**0.519**	***	**0.681**	***	**0.705**	***
Tfh	BCL6	**0.271**	***	**0.360**	**	**0.462**	***
	IL21	**0.163**	**	**NA**	NA	**0.183**	***
T cell exhaustion	PD-1 (PDCD1)	**0.266**	***	**0.358**	**	**0.132**	**
	CTLA4	**0.259**	***	**0.475**	***	**0.123**	0.12
	TIM-3 (HAVCR2)	**0.339**	***	**0.364**	**	**0.330**	***

Cor, *r* value of Spearman's correlatio. *$p < 0.05$; **$p < 0.01$; ***$p < 0.001$.

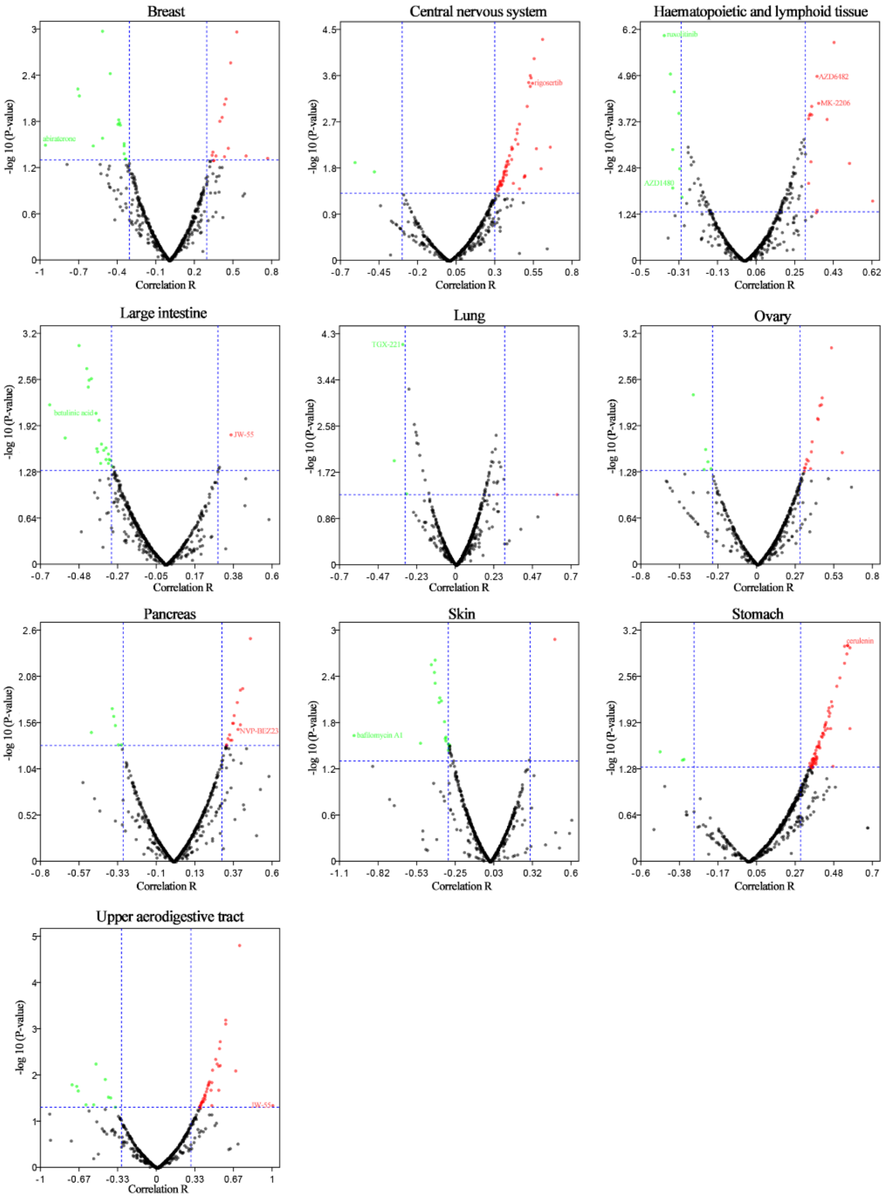

Figure 1. STAT3 expression and drug response correlation in 10 types of cancer cell lines. The volcano plots showed the correlation between STAT3 expression and drugs response across 10 types cancer cell lines by CCLE and CTRP. (The green color meaning the drugs with negative correlation coefficient greater than 0.3, and the red color representing the drugs with positive correlation coefficient greater than 0.3.).
